# Cancer-Associated SF3B1 Mutations Confer a BRCA-Like Cellular Phenotype and Synthetic Lethality to PARP Inhibitors

**DOI:** 10.1158/0008-5472.CAN-21-1843

**Published:** 2022-03-03

**Authors:** Katrina M. Lappin, Eliana M. Barros, Satpal S. Jhujh, Gareth W. Irwin, Hayley McMillan, Fabio G. Liberante, Cheryl Latimer, Melissa J. La Bonte, Ken I. Mills, D. Paul Harkin, Grant S. Stewart, Kienan I. Savage

**Affiliations:** 1 Patrick G Johnston Centre for Cancer Research, Queen's University Belfast, Belfast, United Kingdom.; 2 Institute of Cancer and Genomic Sciences, University of Birmingham, Birmingham, United Kingdom.; 3 Wellcome Sanger Institute, Cambridge, United Kingdom.

## Abstract

Mutations in *SF3B1* have been identified across several cancer types. This key spliceosome component promotes the efficient mRNA splicing of thousands of genes including those with crucial roles in the cellular response to DNA damage. Here, we demonstrate that depletion of SF3B1 specifically compromises homologous recombination (HR) and is epistatic with loss of BRCA1. More importantly, the most prevalent cancer-associated mutation in SF3B1, K700E, also affects HR efficiency and as a consequence, increases the cellular sensitivity to ionizing radiation and a variety of chemotherapeutic agents, including PARP inhibitors. In addition, the SF3B1 K700E mutation induced unscheduled R-loop formation, replication fork stalling, increased fork degradation, and defective replication fork restart. Taken together, these data suggest that tumor-associated mutations in SF3B1 induce a BRCA-like cellular phenotype that confers synthetic lethality to DNA-damaging agents and PARP inhibitors, which can be exploited therapeutically.

**Significance:**

The cancer-associated SF3B1^K700E^ mutation induces DNA damage via generation of genotoxic R-loops and stalled replication forks, defective homologous recombination, and increased replication fork degradation, which can be targeted with PARP inhibitors.

## Abstract (precis)

The cancer-associated SF3B1^K700E^ mutation induces DNA damage via generation of genotoxic R-loops and stalled replication forks, defective homologous recombination, and increased replication fork degradation, which can be targeted with PARP inhibitors.

## Graphical Abstract

**Figure:**
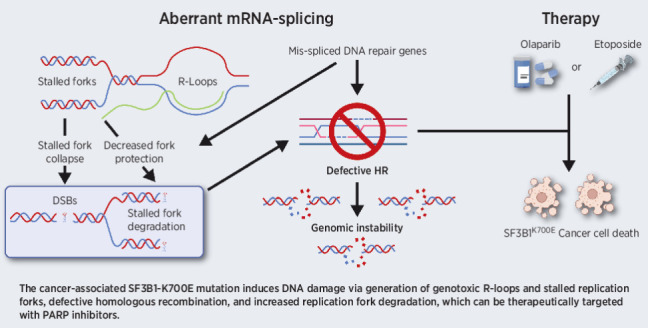

## Introduction

The DNA damage response (DDR) is a highly complex network of pathways that play an essential role in maintaining genome stability and protecting cells from malignant transformation. It is becoming clear that the DDR does not solely focus on regulating cellular pathways linked with DNA metabolism, such as DNA replication and repair, but has also been implicated in controlling a variety of different RNA-regulatory processes, such as transcription, mRNA splicing, RNA export, and nonsense-mediated decay. In relation to this, we previously found that a key DDR protein, BRCA1, forms a DNA damage–inducible complex with several key splicing factors including BCLAF1 and SF3B1, to stimulate the pre-mRNA splicing of a specific set of target genes, which function to facilitate DNA damage and DNA repair (1).

Of all the known spliceosome components, *SF3B1* is the most commonly mutated in human cancers. Mutations in this gene are recurrently observed in myelodysplastic syndromes (MDS) and other myeloid/lymphoid neoplasms, with the heterozygous missense substitution K700E being the most prevalent (2–4). Moreover, *SF3B1* mutations have also been identified in many solid tumor types including uveal melanoma, breast, pancreatic, and prostate cancer (5–9). The role of SF3B1 mutations in disease progression remains controversial, with different roles played in different disease settings. Similarly, the impact of these mutations on disease outcome is also dependent on disease type and/or stage (10).

SF3B1, along with other components of the splicing machinery, aids in the recognition of branch point sequences (BPS) at 3′ splice sites, thereby promoting efficient mRNA splicing (11). As a result, mutation of SF3B1 often results in altered 3′-splice site (A3SS) usage and/or skipped exons (SE) in target genes (11). Therefore, mutation of SF3B1 significantly alters the splicing of thousands of genes with a diverse array of functions. Consequently, the precise mechanism with which these spliceosome mutations influence the initiation and/or progression of disease, or affect the cellular response to treatment, remains unclear (12, 13). Here, we demonstrate that SF3B1 is essential for the correct splicing of several genes whose functions are critical for initiating a cellular response to DNA breaks and replication stress. Critically, we show that the cancer-associated SF3B1^K700E^ mutation causes the accumulation of unscheduled R-loops that triggers replication fork stalling and collapse. Moreover, the inability of cells expressing the SF3B1^K700E^ mutation to repair and restart collapsed forks using HR contributes to the increased genome instability exhibited in these cells. Importantly, we also demonstrate that this aberrant DNA replication and repair can be exploited therapeutically to selectively target tumor cells carrying the SF3B1^K700E^ mutation by using DNA-damaging agents, such as etoposide, or synthetic lethal small-molecule inhibitors, such as PARP inhibitors, which induce HR-dependent mechanisms to repair the genotoxic damage. As such, these data provide the first evidence that tumors with spliceosome mutations can be specifically targeted with clinically relevant therapies currently in use.

## Materials and Methods

### Cell lines

293T (HEK293T; RRID:CVCL_0063) and U2OS (RRID:CVCL_0042) cells were obtained from the Cancer Research UK Cell Repository (London Research Institute, London, United Kingdom). 293T and U2OS cell lines were maintained in DMEM (Sigma-Aldrich) containing 10% FCS. U2OS-DR-GFP cells were a kind gift from Prof. Jeremy Stark (Beckman Research Institute of the City of Hope, Duarte, California). K-562 (RRID:CVCL_0004) cell lines were obtained from DSMZ and maintained in RPMI1640 supplemented with 10% FCS. Polyclonal, stable inducible GFP-RNaseH1 K562 cells were generated via lentiviral infection with pCLX-pTF-R1-DEST-R2-EBR65-RNH1-GFP lentiviral particles and selection was done with 10 μg/mL blasticidin. H2595 (RRID:CVCL_A545), H2591 (RRID:CVCL_A543), REN (RRID:CVCL_M202), and H28 (RRID:CVCL_1555) were cultured in RPMI1640 media supplemented with 10% FCS plus nonessential amino acids for the REN cell line. H2595 has the SF3B1-K700E mutation and the remaining cell lines are wild-type for SF3B1. All cell lines stocks have been authenticated by short tandem repeat profiling and were regularly verified as *Mycoplasma* free.

### Ionizing radiation

Irradiations (IR) were carried out using an X-RAD 225 kV X-ray generator (Precision X-ray Inc.) at a dose rate of 0.591 Gy/minute.

### Clonogenic survival assay

293T and U2OS cells were seeded as single cells at various densities following transfection with siRNAs and 62 hours incubation. Ten hours later, cells were exposed to IR. After 7–14 days, cells were fixed, stained with crystal violet, and colonies counted and the surviving fractions calculated. Clonogenic survival assays performed on the K-562 suspension cell line were done using Methylcellulose base media (HSC002, R&D Systems). Cells were seeded at a density of 10,000 cells per mL, and the compound being tested was added at the desired concentrations and vortexed briefly before plating. After 10 days, Methylcellulose was stained for 16 hours at 37°C with 8 mg/mL of iodonitrotetrazolium chloride in ethanol. Experiments were carried out a minimum of three times, with significant differences in survival assessed by two-way ANOVA, with Tukey multiple comparisons test. Significant differences are indicated by *, *P* < 0.05; **, *P* < 0.01; ***P < 0.001; ****, *P* < 0.0001.

### siRNAs

siRNAs were obtained from QIAGEN. The sequences are listed as follows: SF3B1_5 – TACGAGTTTGCTTGGTCAGAA; SF3B1_7 – GACCGGGAAGATGAATACAAA and siSCR – AGCAGCACGACTTCTTCAAGT. siBRCA1 - ACCATACAGCTTCATAAATAA

### Plasmids

pCMV6-AN-Myc-DDK (PS100016; RRID:SCR_021264) and pCMV6-AN-mRFP (PS100049; RRID: SCR_021265) were purchased from Origene. Inducible GFP-RNAseH1 plasmid was generated via restriction cloning an RNH1-GFP cassette from pEGFP‐RNASEH1, a gift from Andrew Jackson and Martin Reijns (RRID: Addgene_108699) using *Bgl*II + *Not*I and cloning into the *Bam*H1 *Not*I sites of pENTR1A (RRID:SCR_021263). The RNH1-GFP cassette was then transferred to the pCLX-pTF-R1-DEST-R2-EBR65 lentiviral plasmid, a gift from Patrick Salmon (RRID:Addgene_45952) via an LR (recombination reaction between attL and attR sites) recombination reaction, creating a single vector that expresses the Tet-repressor and blasticidin resistance gene under the control of a constitutive promoter and containing the RNHA1-GFP cassette under the control of a Tet-repressor regulated promoter. SF3B1-K700E and RNAseH1 D210N mutations were created by site-directed mutagenesis.

### Western blotting

Whole-cell extracts (WCE) were prepared by lysing cells in two volumes of Lysis buffer (50 mmol/L Tris pH 7.4, 150 mmol/L NaCl, 5 mmol/L EDTA, 50 mmol/L HEPES, 1% v/v Triton-X100, 0.1% v/v SDS). Thirty to 60 μg of WCE was resolved on 4%–12% Bolt Gels (Thermo Fisher Scientific). Protein was transferred to nitrocellulose membrane (Thermo Fisher Scientific) and blotted for SF3B1 (Bethyl Laboratories: A300–996A; RRID:AB_805834)), Flag M2 (Sigma-Aldrich: F3165; RRID:AB_439685), mRFP (Origene:TA180093; RRID:AB_2622287) and γ-tubulin (GTU-88:Sigma Aldrich; RRID:AB_532292), ATM (2C1:Abcam; RRID:AB_368161), BRCA2 (OP95:Millipore; RRID: AB_2067762), ATR (N19:SCBT; RRID:AB_630893), Mre11 (4895:Cell Signaling Technology; RRID:AB_2145100), BRCA1 (D-9:SCBT; RRID:AB_626761), RAD51 (SCBT:3C10/sc-53428; RRID:AB_630180), γH2AX (Millipore:JBW301/05–636; RRID:AB_309864), GAPDH (Sigma: HPA040067; RRID:AB_10965903), vinculin (Abcam:ab219649; RRID: AB_2819348), β-actin (SCBT:C4; RRID: AB_2714189), and GFP (D5.1:Cell Signaling Technology; RRID:AB_1196615).

### Immunofluorescence staining and quantification of DNA damage/repair markers

Following treatment and appropriate recovery as indicated, cells were fixed in 4% PFA/PBS and then permeabilized in 0.4% Triton X-100/PBS followed by blocking in 3% BSA/PBS. Cells were stained with γH2AX (Millipore: JBW301/05–636; RRID:AB_309864), 53BP1 (Novus Biologicals: NB100–304; RRID:AB_10003037), or RAD51 (SCBT:3C10/sc-53428; RRID:AB_630180) primary antibodies and anti-mouse AlexaFluor 488/568 (Thermo Fisher Scientific) secondary antibodies. Cells were counterstained with DAPI to identify cellular nuclei. Representative images were acquired using a Nikon Eclipse Ti-S fluorescence microscope, with 60× objective or using an ArrayScan XTI High-content Imaging system with 40× objective (Thermo Fisher Scientific) and using the spot detection algorithm for foci/cell scoring. Cells were scored as positive or negative for unrepaired DNA damage as indicated in figures, with a minimum of 100 cells per condition scored with each condition per experiment carried out a minimum of three times independently. Significant differences between quantified data were assessed using multiple two-tailed *t* tests, with *post hoc* Holm–Šidák multiple comparisons tests. Significant differences are indicated by *, *P* < 0.05; **, *P* < 0.01; ***, *P* < 0.001; ****, *P* < 0.0001.

### R-loop detection

Cells were fixed in 4% PFA/PBS followed by permeabilization with 0.4% Triton-X100 and blocking with 3% BSA/PBS. Cells were stained with S9.6 (Kerafast:ENH001; RRID:AB_2687463), and anti-nucleolin (Abcam:ab50279; RRID:AB_881762) primary antibodies and AlexaFluor 488/568 (Thermo Fisher Scientific) secondary antibodies. Cells were counterstained with DAPI before being imaged using a Nikon Eclipse Ti-S fluorescence microscope with 60× objective with quantification of nuclear S9.6 staining carried out by measuring mean fluorescence intensity within DAPI stained regions using imageJ (RRID:SCR_003070). Regions of nucleolin staining were subtracted from final quantification. Alternatively, GFP-RNH1^D210^ was induced for 24 hours with 1 μg/mL doxycycline, after which, nonbound GFP-RNH1^D210^ was extracted with extraction buffer (20 mmol/L Hepes, 20 mmol/L NaCl, 5 mmol/L MgCl_2_, 1 mmol/L ATP, 0.5% NP40) at 4°C for 2 minutes before fixation with 4% PFA. Fixed cells were then stained with DAPI and nuclear GFP intensity quantified via high-content imaging using a Thermo ArrayScan XTI. Significant differences between quantified data were assessed using multiple two-tailed *t* tests, with *post hoc* Holm–Šidák multiple comparisons tests. Significant differences are indicated by *, *P* < 0.05; **, *P* < 0.01; ***, *P* < 0.001; ****, *P* < 0.0001.

### DR-GFP homologous recombination and NHEJ-GFP reporter assays

U2OS-DR-GFP cells were a kind gift from Prof Jeremy Stark (Beckman Research Institute of the City of Hope, Duarte, CA) and DR-GFP assays carried out as described previously (14).

The pimEJ5GFP non-homologous end joining (NHEJ) reporter plasmid, a kind gift from Jeremy Stark (Addgene plasmid # 44026; http://n2t.net/addgene:44026; RRID:Addgene_44026) was transfected into SF3B1^WT^ and SF3B1^K700E^ K-562 cells using the Amaxa 4D-Nucleofector as per the recommended protocol for K562 cells, after which, stably transfected cells were selected with puromycin (1 μg/mL). Cells were then infected with the I-SceI–carrying adenovirus AdNGUS24i (a gift from Frank Graham, McMaster University, Hamilton, Ontario, Canada) at an estimated multiplicity of infection of 10. NHEJ was assessed using flow cytometry to quantify GFP-positive cells 96 hours after AdNGUS24i infection. GFP positivity was assessed using flow cytometry with a minimum of 10,000 cells per condition with each condition/experiment carried out a minimum of three times. Significant differences between quantified data were assessed using multiple two-tailed *t* tests, with *post hoc* Holm–Šidák multiple comparisons tests. Significant differences are indicated by *, *P* < 0.05; **, *P* < 0.01; ***, *P* < 0.001; ****, *P* < 0.0001.

### Replication fork speed, restart, and fork degradation analysis

SF3B1^WT^ and SF3B1^K700E^ K-562 cells were grown in suspension cultures. 8.0 × 10^5^ cells were plated into 6-well culture dishes 24 hours prior to any treatments and nascent DNA labeling. Untreated cells were pulse labeled with 25 μmol/L CldU added directly to cell culture medium. Cells were transferred to a universal with 18 mL of culture medium containing 250 μmol/L IdU and washed twice in medium containing 250 μmol/L IdU, before being resuspended in medium containing 250 μmol/L IdU, transferred to a 6-well culture dish, and incubated at 37°C for 20 minutes. The cells were then transferred to a universal containing 18-mL ice-cold PBS and spun at 1,400 rpm for 4 minutes. The supernatant was discarded, the cells were resuspended in 200 μL ice-cold PBS, and volume was adjusted to ensure a final concentration of 5.0 × 10^5^ cells per mL. For replication fork restart, 8.0 × 10^5^ cells were grown for 24 hours and pulsed labeled with 25 μmol/L CldU as above. Cells were transferred into a universal containing cell culture medium with 2 mmol/L hydroxyurea (HU), the cells were spun at 1,400 rpm for 4 minutes, the supernatant was discarded. The cell pellet was washed in 20 mL HU-containing media and pelleted as before. The supernatant was discarded and cells resuspended in 2 mL of HU-containing media and incubated at 37°C for 2 hours. Cells were then pulse labeled with 250 μmol/L IdU and harvested for DNA fiber analysis as above. For fork degradation, 8.0 × 10^5^ cells were grown for 24 hours and pulsed labeled with 25 μmol/L CldU for 20 minutes, followed by 250 μmol/L IdU for 20 minutes as above, after which, cells were washed and treated with 4 mmol/L HU for 5 hours. DNA fiber analysis was carried out as described previously (15). For ongoing forks, new origins, and stalled forks, four independent experiments were performed with >150 CldU-labeled forks per experiment analysed. Significant differences in fork types were assessed using two-tailed *t* tests. *, *P* < 0.05; **, *P* < 0.01; ***, *P* < 0.001. For fork speed and degradation, three independent experiments were carried out with >150 fibers scored per experiment. Significant differences in fork speed were assessed using unpaired Mann–Whitney *U* test (****, *P* < 0.0001).

### *In vivo* tumor growth and treatment

All animal studies within this manuscript were approved by the Queen's University Belfast Animal Welfare and Ethics Review Board (AWERB) and the Northern Ireland Department of Health. SF3B1^WT^ and SF3B1^K700E^ K-562 cells were maintained at an exponential growth phase. Cell viability was determined using Trypan blue exclusion assay (minimum of 98% viability) prior to preparation for injection. Each mouse (5–6 weeks old; athymic BALB/c) received a single subcutaneous injection into the flank of a hind leg containing 1 × 10^6^ cells per 100 μL of Matrigel suspension (100 μL/injection). Tumors were measured using a digital caliper until an average size of 100 mm^3^ was reached. Animals were then randomized into appropriate group sizes of average tumor size and treated with either vehicle (*n* = 6) for 5 days per week, 50 mg/kg olaparib (*n* = 6) for 5 days/week, or 20 mg/kg etoposide (*n* = 6) 1 day per week. Animal weight was assessed prior to each treatment and tumor volume was assessed three times weekly. Animals were humanely euthanized when maximum tumor size was reached (1,000 mm^3^). Fold change from initiation of treatment was calculated for each tumor per mouse, with mean tumor volumes per group reported. Significant differences in tumor volume were assessed at day 28 using multiple two-tailed *t* tests, with *post hoc* Holm–Šidák multiple comparisons tests. Multiple comparisons *P*_adj_ values have been reported.

### Sister chromatid exchange assay

8.0 × 10^5^ cells were seeded and grown for 24 hours before 10 μmol/L BrdU was added for 2 passages (48 hours). Cells were then treated with 2 Gy IR and allowed to recover for 36 hours, after which, 0.2 μg/mL colcemid (KaryoMax) was added and cells incubated for 12 hours. Cells were then pelleted and resuspended gently in 10 mL prewarmed 0.075 mol/L KCl incubated at 37°C for 20 minutes, following which, 2 mL of Carnoy's fixative (3:1 methanol/acetic acid) was mixed in and the cells pelleted. Cells were resuspended in 10 mL Carnoy solution and cells left overnight at 4°C. Cells were then pelleted and resuspended in 2 mL Carnoy solution and then dropped onto wet slides prewarmed at 45°C. Slides were then incubated in 10 μg/mL Hoescht for 20 minutes in the dark, followed by incubation in 20xSSC buffer for 5 minutes. 1 mL of 20xSSC was added to each slide and a coverslip placed on top before slides were placed under a UV lamp for 1.5 hours. Following this, slides were placed in 20xSSC buffer for 1 hour at 60°C. The slides were washed in water before being stained with Giemsa (1:20 dilution in water) for 15 minutes, washed again in water for 3 minutes, and left to dry overnight. Coverslips were mounted with Cytoseal and imaged and exchanges counted using a Nikon Eclipse Ti-S microscope. Quantification of sister chromatid exchanges was carried out from three independent experiments with ≥50 spreads were scored/experiment. Significant differences in the number of SCEs were assessed using two-tailed *t* tests. ***, *P* < 0.001; ****, *P* < 0.0001.

### RNA-seq and data analysis

Total, nuclear, and cytoplasmic RNA preparations, RNA sequencing (RNA-seq), and downstream data analysis, including differential splicing analysis using dSpliceType was carried out as described previously (16). Ingenuity Pathway Analysis (IPA; QIAGEN) of A3SS and SE data outputs from dSpliceType was carried out using IPA core expression analysis with default settings (IPA Version: 68752261, Build: ing_emerald).

### Data availability statement

RNA-seq data is available in the ArrayExpress (EMBL-EBI) repository, under accession E-MTAB-7192.

## Results

### SF3B1 is required for an efficient DDR

**Figure 1.**
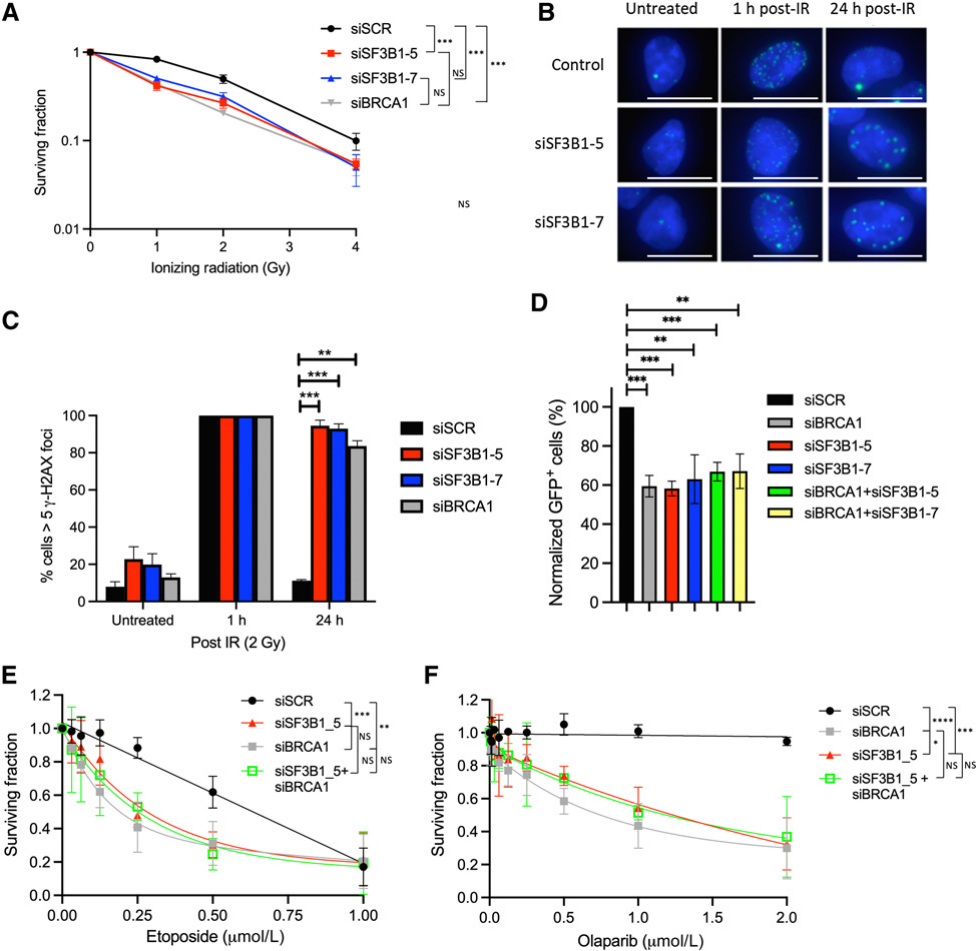
SF3B1 is required for an efficient DDR. **A,** Clonogenic survival assays demonstrating that depletion of SF3B1 (two independent siRNAs) induces sensitivity to IR in 293T cells. Mean surviving fraction of three independent experiments is plotted ± SEM. **B,** Representative immunofluorescence staining of γH2AX-marked DNA damage in untreated U2OS cells depleted of SF3B1, and 1 and 24 hours following 2 Gy IR. White scale bar, 20 μm. **C,** Quantification of three independent experiments described above (≥200 cells were scored/experiment). Mean percent cells containing ≥5 γH2AX foci is plotted ± SEM. **D,** Quantification of GFP-positive cells by flow cytometry from a DR-GFP reporter cassette stably integrated in U2OS cells, to assess HR. Mean % GFP-positive cells normalized to siSCR-transfected cells is plotted ± SEM. **E** and **F,** Long-term cellular survival assays demonstrating that depletion of SF3B1 (two independent siRNAs) induces sensitivity to etoposide and olaparib in U2OS cells and that SF3B1 and BRCA1 are epistatic. Mean surviving fraction of three independent experiments is plotted ± SEM. *, *P* < 0.05; **, *P* < 0.01; ***, *P* < 0.001; ****, *P* < 0.0001; NS, nonsignificant.

We have previously shown that the BRCA1 mRNA-splicing complex, which includes SF3B1, facilitates DNA repair, in part through its ability to promote the efficient splicing of genes involved in the DNA damage response (1). Given that SF3B1 is the most highly mutated member of this complex in a variety of cancers, we hypothesized that loss or mutation of this subunit would result in a DNA repair defect akin to what is observed in BRCA1-mutant tumors. Therefore, to assess this, we initially determined whether SF3B1 depletion would have a deleterious impact on cell survival following the induction of DNA double-strand breaks (DSB) using two independent siRNAs. In keeping with our hypothesis, depletion of SF3B1 resulted in an increased sensitivity to IR-induced DNA damage comparable to BRCA1 (Fig. 1A; Supplementary Fig. S1A; ref. 1).

To investigate this further, we assessed the efficiency of DNA repair using γH2AX as a marker of DSBs in control and SF3B1-depleted cells following exposure to IR. Indeed, depletion of SF3B1 resulted in a profound defect in the resolution of γH2AX marked DSBs 24 hours postirradiation, indicative of defective DNA repair in these cells (Fig. 1B and C; Supplementary Fig. S1B). DNA DSBs are primarily repaired through one of two mechanisms—NHEJ or homologous recombination (HR). As mRNA splicing has previously been implicated in HR (1), we used the well-characterized DR-GFP HR reporter assay, to evaluate whether the DNA repair deficiency observed in SF3B1-depleted cells was caused by a defect in HR (17, 18). Using this system, we observed a marked reduction in the capacity of SF3B1-depleted cells to carry out HR in a similar fashion to BRCA1 depletion (Fig. 1D; Supplementary Fig. S1B). Indeed, combined depletion of both BRCA1 and SF3B1 did not result in increased HR deficiency, suggesting that these two proteins function within the same HR-dependent DNA repair pathway (Fig. 1D; Supplementary Fig. S1B).

HR-deficient cells are known to be sensitive to the DSB-inducing agent etoposide and also to the PARP inhibitor olaparib, through a synthetic lethal interaction. Therefore, we also investigated the impact of SF3B1 depletion on cellular survival following treatment with these agents. In keeping with our previous findings, SF3B1 depletion led to a significant decrease in cell survival in response to both etoposide and olaparib, suggesting that the role of this protein in HR is crucial to promote cellular survival following DNA damage (Fig. 1E and F). In line with the results above, double depletion of SF3B1 and BRCA1 did not further sensitize these cells to either agent again, suggesting that deficiencies of SF3B1 and BRCA1 are epistatic (Fig. 1E and F).

### The cancer-associated mutation SF3B1^K700E^ promotes abnormal DNA repair and sensitivity to etoposide and olaparib

It is well known that cancer associated *SF3B1* mutations, which are almost exclusively missense mutations, do not result in the abrogation of SF3B1 expression, but rather change how SF3B1 recognizes 3′ splice sites, often leading to cryptic splice site usage, exon skipping and intron retention, which results in disruption of target gene open reading frames (11, 16). Considering this, we generated an isogenic K562 cell line pair containing the SF3B1^K700E^ mutation and a parental/wild-type SF3B1^WT^ counterpart using a CRISPR/Cas9 gene editing system (16). Previous work from our lab and other groups has demonstrated a clear link between SF3B1-directed mRNA splicing and export, where efficient splicing coordinates the export of certain RNAs (14, 16, 19). To functionally validate the SF3B1^K700E^ isogenic model, we performed RNA-seq on total RNA extracted from the K562 SF3B1^K700E^ and SF3B1^WT^ cell lines, or RNA derived from nuclear and cytoplasmic fractions. In line with our previous reports, we identified a significant increase in the amount of nuclear mRNA species and a corresponding decrease in cytoplasmic mRNA levels in the SF3B1^K700E^ cells when compared with the SF3B1^WT^ cells, indicative of defective splicing and mRNA export in SF3B1^K700E^ cells (16). In keeping with this, we observed significantly deregulated mRNA splicing, particularly 3′alternative splice site (3ASS) usage and skipped exon (SE), in a large number of genes in SF3B1^K700E^ cells compared with their SF3B1^WT^ counterparts (Supplementary Table S1; ref. 16).

**Figure 2.**
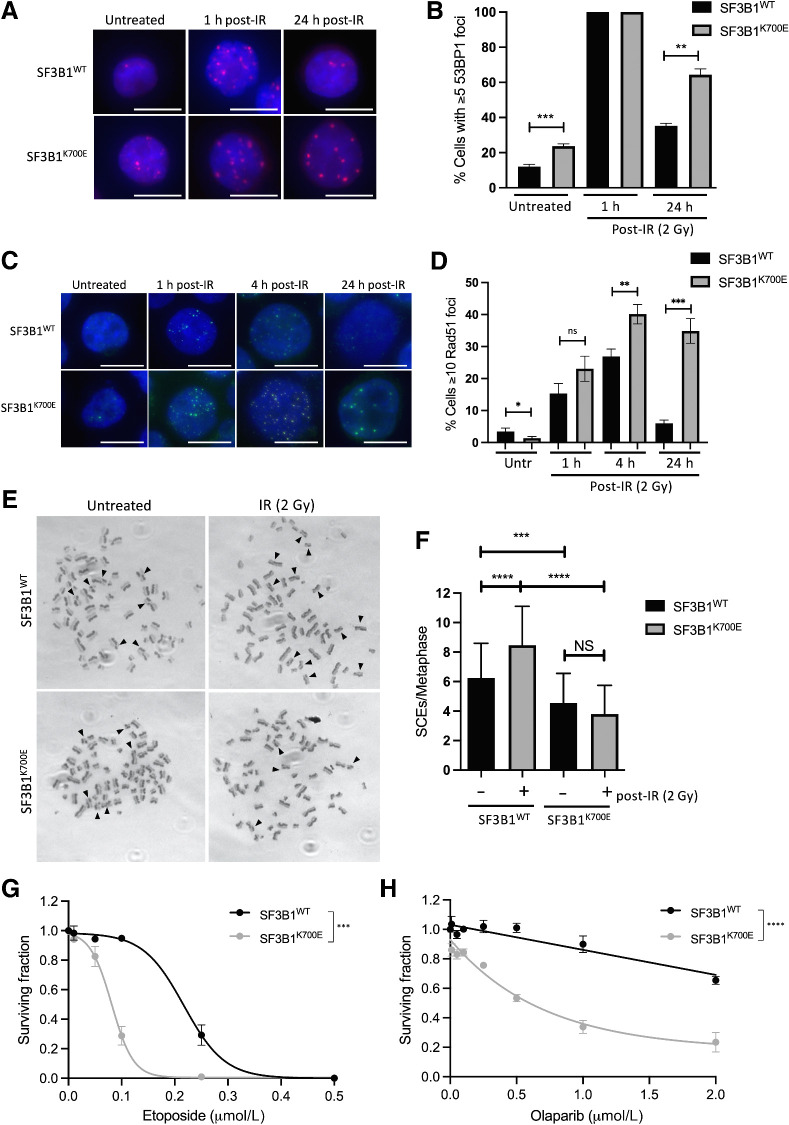
The cancer-associated mutation SF3B1^K700E^ promotes abnormal DNA repair and sensitivity to etoposide and olaparib. **A,** Representative immunofluorescence staining of 53BP1 marked DNA damage in untreated SF3B1^WT^ and SF3B1^K700E^ K562 cells and 1 and 24 hours following 2 Gy IR. White scale bar, 20 μm. **B,** Quantification of 53BP1 foci in cells described in **A** from three independent experiments (≥100 cells were scored/experiment). Mean percentage of cells containing ≥5 53BP1 foci is plotted ± SEM. **C,** Representative immunofluorescence staining of Rad51 marked DNA damage in untreated SF3B1^WT^ and SF3B1^K700E^ K562 cells and 1, 4, and 24 hours following 2 Gy IR. White scale bar, 20 μm. **D,** Quantification of Rad51 foci in cells described in **C** from three independent experiments (≥100 cells were scored/experiment). Mean percentage of cells containing ≥10 Rad51 foci is plotted ± SEM. **E,** Representative SCEs in metaphase spreads from untreated and treated (IR; 2 Gy, 24 hours) SF3B1^K700E^ and SF3B1^WT^ cells. Arrows, SCEs. **F,** Quantification of SCEs from three independent experiments described above (≥50 spreads were scored/experiment). Mean number of SCEs for each condition is plotted ± SEM. **G** and **H,** Clonogenic survival assays in SF3B1^K700E^ and SF3B1^WT^ cells, treated with increasing doses of etoposide (**G**) or olaparib (**H**). Mean surviving fraction ± SEM from three independent experiments plotted. *, *P* < 0.05; **, *P* < 0.01; ***, *P* < 0.001; ****, *P* < 0.0001; NS, nonsignificant.

To assess the biological impact of this deregulated splicing and export, we performed IPA on genes with 3ASS and SE in SF3B1^K700E^ cells. Consistent with a role for SF3B1 in DNA repair, this analysis identified DNA replication, recombination and repair as one of the key deregulated pathways in cells expressing mutant SF3B1 (Supplementary Fig. S1C; Supplementary Table S2). Intriguingly, while we did not observe any appreciable changes in the expression of key HR proteins such as BRCA1, Rad51, ATM, ATR, Mre11, and BRCA2 in SF3B1-mutant cells (Supplementary Fig. S1D), as outlined above, we did observe A3SS usage and SEs in a large number of genes involved in HR, suggesting that SF3B1 mutation may have a polygenic effect on HR. Taken together with the role for SF3B1 in DSB repair highlighted above, we next tested the impact of the cancer associated SF3B1^K700E^ mutation on DSB repair. Indeed, the SF3B1^K700E^ cells were unable to resolve 53BP1 or γH2AX marked DSBs at late times postirradiation compared with their SF3B1^WT^ counterpart (Fig. 2A and B; Supplementary Fig. S2A). A similar DSB repair defect was observed with ectopic expression of SF3B1^K700E^ in SF3B1 wild-type cells and a mesothelioma-derived cell line harboring the SF3B1^K700E^ mutation (H2595) compared with a matched SF3B1 wild-type control mesothelioma-derived line (H2591; Supplementary Fig. S2B–S2D). In addition, using the DR-GFP HR reporter, HR-directed repair was impaired in U2OS cells transfected with ectopic SF3B1^K700E^ compared with those transfected with SF3B1^WT^ (Supplementary Fig. S2E). Intriguingly, we also observed increased 53BP1 foci in SF3B1^K700E^ cells compared with SF3B1^WT^ cells, even in the absence of DNA damage, suggesting increased basal damage in these cells (Fig. 2A and B). Because our IPA analysis also identified the deregulation of some cell-cycle genes in SF3B1^K700E^-expressing cells, we assessed cell-cycle progression in unperturbed SF3B1^WT^ and SF3B1^K700E^ cells following DNA damage. This analysis demonstrated no difference in cell-cycle profiles between SF3B1^WT^ and SF3B1^K700E^ cells, indicating that SF3B1^K700E^ specifically compromises HR without affecting cell-cycle progression (Supplementary Fig. S2F).

To directly determine the impact of the SF3B1^K700E^ mutation on HR, we assessed the formation and resolution of Rad51 foci, a key marker of HR function, in our isogenic models following IR-induced DNA damage. Intriguingly, unlike cells lacking BRCA1, SF3B1^K700E^ cells exhibited a normal ability to recruit Rad51 to sites of DSBs, indicating a functional BRCA1/Rad51 pathway. Interestingly, however, Rad51 foci persisted in the SF3B1^K700E^ cells at the 24 hours timepoint postirradiation, indicative of a defect in the later stages of HR following Rad51 loading and nucleofilament formation (Fig. 2C and D). Similar results were observed with the DSB-inducing agent etoposide (Supplementary Fig. S2G). Given that HR occurs during S/G_2_ phase, we confirmed that these persistent Rad51 foci following IR were restricted to S-phase cells using EdU labeling (Supplementary Fig. S2H). In keeping with this, both the increased 53BP1 foci in unperturbed SF3B1^K700E^ cells, as well as unresolved 53BP1 foci 24 hours following IR, predominantly occurred in EdU-positive cells, suggesting that these DSBs arise and/or fail to be repaired in S-phase (Supplementary Fig. S2H). To examine the impact of SF3B1 mutation on HR further, we assessed sister chromatid exchanges (SCE), an end product of a proportion of HR-mediated repair events. Indeed, in both unperturbed cells, and following IR, we observed significantly decreased SCEs in SF3B1^K700E^ cells, compared with wild-type cells (Fig. 2E and F). Taken together, these data suggest that SF3B1^K700E^ mutation affects the ability of cells to resolve recombination intermediates.

Importantly, HR defects consistently induce sensitivity to DNA-damaging chemotherapeutics, such as etoposide, and to PARP inhibition, through synthetic lethality. Therefore, we assessed the sensitivity of SF3B1^K700E^ cells to etoposide and the clinically relevant PARP inhibitor olaparib. In keeping with our previous results, SF3B1^K700E^ cells were significantly more sensitive to these agents than their wild-type counterpart (Fig. 2G and H). In addition, the SF3B1^K700E^-mutant cell line H2595 was also significantly more sensitive to olaparib and etoposide, compared with the matched SF3B1^WT^ cell line, H2591 (Supplementary Fig. S3A).

To ensure that the increased sensitivity of SF3B1-mutant cells to etoposide was specifically caused by a defect in HR, we also assessed NHEJ function in SF3B1^WT^ and SF3B1^K700E^ cells using a fluorescent NHEJ reporter that measures NHEJ mediated joining of two tandem endonuclease cut sites, resulting in the juxtaposition of an active promoter upstream of a functional GFP gene (Supplementary Fig. S3B; ref. 20). In keeping with a HR defect, which often results in increased NHEJ-mediated DSB repair (21), SF3B1^K700E^ cells had increased DNA end-joining compared with SF3B1^WT^ isogenic cells (Supplementary Fig. S3C).

### SF3B1^K700E^ mutation induces unscheduled R-loops, stalled replication forks, and reduced replication fork protection and restart

**Figure 3.**
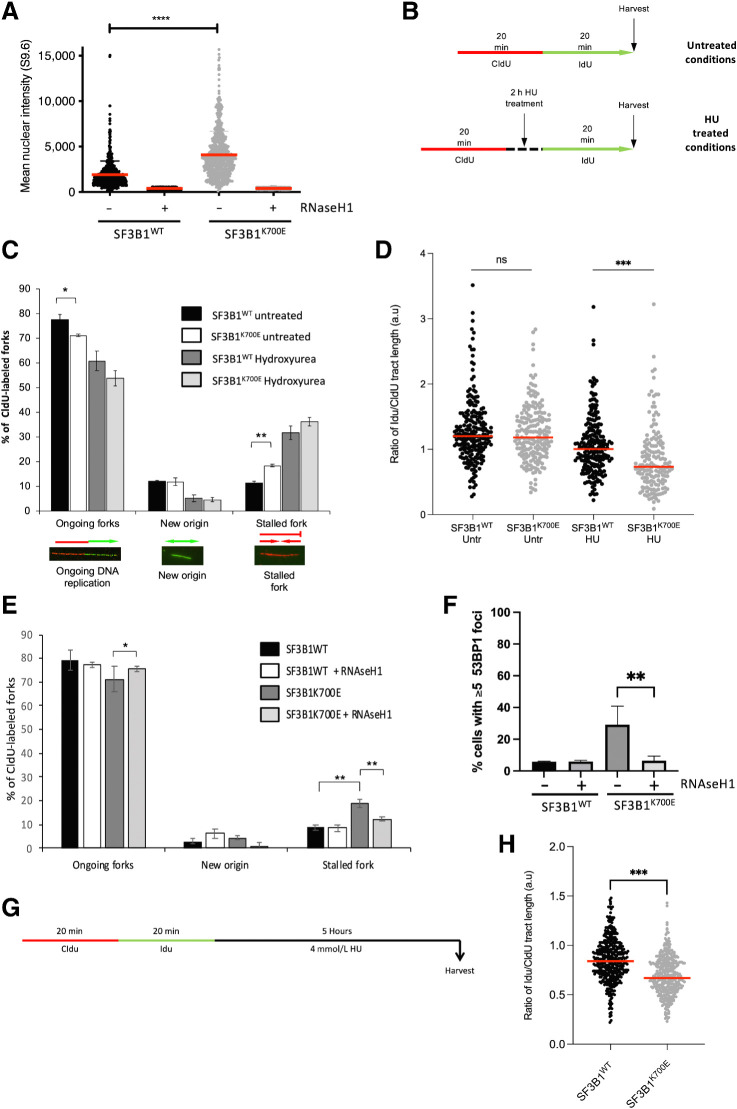
SF3B1^K700E^ mutation induces unscheduled R-loops, stalled replication forks, and reduced replication fork restart. **A,** Quantification of nuclear fluorescence intensity from SF3B1^K700E^ and SF3B1^WT^ cells stained with the DNA:RNA hybrid-specific antibody (S9.6). RNaseH1 treatment was used to control for DNA:RNA hybrid staining. Mean fluorescence intensity of individual nuclei (>100 cells/experiment) from three independent experiments is plotted ± SEM. **B,** Schematic representation of replication fork labeling assay. Untreated cells were labeled with CldU for 20 minutes, after which, CldU was removed and the cells washed before labeling with IdU for a further 20 minutes. Treated cells were labeled with CldU for 20 minutes and CldU removed; cells were then incubated with 2 mmol/L HU treatment for 2 hours. The treatment was then washed off and cells labeled with IdU for 20 minutes before harvesting. **C,** Quantification of ongoing forks, new origins, and stalled forks scored in CldU-labeled forks in untreated or HU-treated (2 mmol/L, 2 hours) SF3B1^WT^ and SF3B1^K700E^ cells. Representative images of each fork type are shown. Mean percentage of each fork type ± SEM is plotted from four independent experiments (>150 CldU-labeled forks/experiment). **D,** Replication fork speed in untreated or HU-treated (2 mmol/L, 2 hours) SF3B1^WT^ and SF3B1^K700E^ cells. Data points represent fork speed from four independent experiments, with median fork speed for each condition indicated (red line). **E,** Quantification of ongoing forks, new origins, and stalled forks scored in CldU-labeled forks in untreated SF3B1^WT^ and SF3B1^K700E^ cells with and without induction of RNAseH1 for 24 hours prior to CldU/IdU labeling. Mean percentage of each fork type ± SEM is plotted from three independent experiments (>250 CldU-labeled forks/experiment). **F,** Quantification of 53BP1 foci in untreated SF3B1^WT^ and SF3B1^K700E^ cells with and without induction of RNAseH1 (1 μg/mL doxycycline, 24 hours). Mean percentage of cells containing ≥5 53BP1 foci is plotted ± SEM. **G,** Schematic representation of replication fork labeling assay. Cells were sequentially labeled with CldU and IdU for 20 minutes, respectively, the IdU washed off, and the cells incubated with 4 mmol/L HU for 5 hours prior to harvesting. **H,** Fork degradation in SF3B1^WT^ and SF3B1^K700E^ cells. Cells were labeled and treated as outlined in **G**. DNA was visualized with antibodies to CldU and IdU, and the length of CldU and IdU tracts measured at ongoing replication forks. A ratio was calculated of IdU to CldU track lengths. Plots depict the average ratios of IdU:CldU tract lengths from three independent experiments, with >150 ongoing replication forks measured per experiment. Median IdU:CldU in each cell line is indicated (red line). *, *P* < 0.05; **, *P* < 0.01; ***, *P* < 0.001; ****, *P* < 0.0001; ns, nonsignificant.

Our data clearly suggests a role for SF3B1 in the DDR, particularly in HR-mediated DNA repair following DNA damage. However, we have also observed increased DSBs and reduced HR in unperturbed SF3B1^K700E^ cells. Importantly, a number of cancer-associated mutations in mRNA splicing factors that disrupt splicing efficiency and/or splice-site recognition have been shown to induce R-loops, which can themselves lead to DNA damage, particularly when encountered by an advancing replication fork, resulting in replication fork stalling (22–26). Given that HR-mediated repair is required for the resolution of stalled replication forks and subsequent replication fork restart, we hypothesized that the SF3B1^K700E^ mutation may induce R-loops, which increases genome instability by stalling and collapsing replication forks. To test this, we assessed R-Loop formation using the well-characterized S9.6 antibody, which detects RNA:DNA hybrids. Indeed, quantification of S9.6 staining in our isogenic cell model revealed significantly more R-Loops in SF3B1^K700E^ cells compared with their SF3B1^WT^ counterpart (Fig. 3A; Supplementary Fig. S4A). We validated these findings by treating the cells with RNaseH1 (an R-loop–specific nuclease; Fig. 3A). We also stably introduced a doxycycline-inducible, GFP-tagged RNaseH1 into these cell lines, which has been catalytically inactivated via D210N substitution. RNaseH1^D210N^ is capable of binding but not resolving R-loops (27). This results in the accumulation of GFP-RNaseH1^D210N^ at R-loops, which can be visualized and quantified using high-content fluorescence microscopy as an alternative method for quantifying R-loops (27). Importantly, this system also revealed significant accumulation of R-loops in SF3B1^K700E^ cells compared with their SF3B1^WT^ counterpart (Supplementary Fig. S4B and S4C).

To assess whether the increased R-loops present in the SF3B1^K700E^-mutant expressing cell line affects DNA replication, we examined replication fork dynamics using the DNA fiber assay (Fig. 3B). Notably, this analysis revealed a significantly higher level of stalled replication forks in unperturbed SF3B1^K700E^ compared with SF3B1^WT^ cells, with a concomitant reduction in ongoing replication fork structures (Fig. 3C). Interestingly, however, the level of stalled replication forks induced following exposure to HU was comparable between the SF3B1 WT- and K700E-expressing cell lines, suggesting that the SF3B1^K700E^ mutation does not affect replication fork stability following the induction of replication stress by HU. Moreover, the suppression of new origin firing following exposure to HU was also similar between the WT and mutant cell line, indicative that the ATR-dependent intra-S phase checkpoint is unaffected (Fig. 3C). Despite this, we did observe a significant reduction in the efficiency of replication fork restart in SF3B1^K700E^ cells following release from a transient HU-induced replication block, in the absence of any obvious alterations in the basal levels of replication speed (Fig. 3D). These data are in keeping with the requirement for an intact HR pathway to facilitate the repair and restart of stalled replication forks (28).

To test whether the increased R-loops observed in SF3B1^K700E^ cells were responsible for the increased spontaneously stalled forks, we again used the DNA fiber assay to quantify the levels of stalled forks in these cells following the induction of WT GFP-RNaseH1. Notably, the induction of RNaseH1 completely rescued the increased spontaneously stalled forks observed in SF3B1^K700E^ cells, to levels comparable with the SF3B1^WT^ cells (Fig. 3E; Supplementary Fig. S4C). Similarly, the induction of RNaseH1, also reduced the elevated levels of spontaneous DSBs, as measured by the presence of 53BP1 foci, in SF3B1^K700E^ cells (Fig. 3F). Combined, these findings indicate that the increased genome instability resulting from the SF3B1^K700E^ mutation is caused by unresolved R-loops triggering replication fork stalling and collapse into DSBs. We next tested whether the presence of increased R-loops in SF3B1^K700E^ cells altered the sensitivity of these cells to etoposide or olaparib by performing clonogenic survival assays in the presence of doxycycline-induced RNAseH1. RNAseH1 induction in these cells did not alter sensitivity to olaparib or etoposide, indicating that the induction of DNA damage, and subsequent DNA repair and cellular survival following treatment with these agents, is not dependent on R-loops (Supplementary Fig. S4D).

Because we had previously shown that SF3B1^K700E^ cells displayed an increased sensitivity to etoposide and olaparib, we utilized the DNA fiber assay to determine whether this requirement for SF3B1-dependent repair and restart of damaged replication forks could underlie the hypersensitivity of the mutant cells to etoposide and olaparib. In keeping with this notion, the mutant SF3B1 cells displayed a striking reduction in replication fork progression following exposure to either etoposide or olaparib, when compared with their SF3B1^WT^ counterpart (Supplementary Fig. S4E).

Finally, it is known that following replication arrest, several proteins, including BRCA1 and BRCA2, are required for preventing uncontrolled nucleolytic degradation of damaged forks and loss of this function contributes to the hypersensitivity of HR-deficient tumors to replication stalling genotoxic agents. Given that we have shown that SF3B1^K700E^ cells display a BRCA-like phenotype, we investigated whether mutations in SF3B1 could also compromise replication fork stability. This was carried out by dual labeling of newly replicated DNA with equal sequential short pulses of CldU and ldU, followed by a prolonged HU-induced replication fork arrest (Fig. 3G). Interestingly, similar to BRCA1-deficient cells, we observed increased fork degradation in SF3B1^K700E^ cells compared with their SF3B1^WT^ counterparts (Fig. 3H). In keeping with this observation, SF3B1^K700E^ cells failed to properly recruit Rad51 to replication forks stalled following exposure to HU or olaparib (Supplementary Fig. S4F and S4G). Taken together, our data demonstrate that the common tumor-associated mutation in SF3B1, K700E, recapitulates many of the cellular deficits that are characteristic of loss of BRCA1/2.

### SF3B1^K700E^-associated xenografts are sensitive to etoposide and olaparib

**Figure 4.**
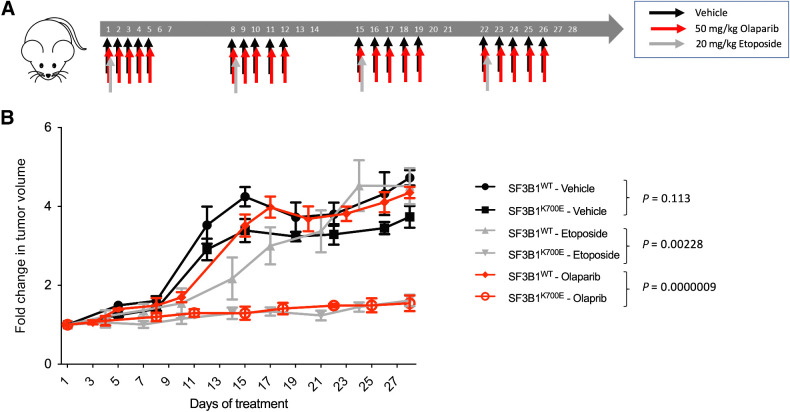
SF3B1^K700E^-associated xenografts are sensitive to etoposide and olaparib. **A,***In vivo* treatment schedule. Mice were randomly assigned to three different treatment groups: vehicle, olaparib, or etoposide. Treatments were administered as indicated. **B,** Fold change in tumor volume in mice from the different treatment groups described above. Mean fold change in tumor volumes from the 6 mice in each treatment condition is plotted ± SEM.

Because our data indicate that the SF3B1^K700E^ mutation gives rise to an HR defect, we hypothesized that this could be exploited therapeutically to treat tumors that have acquired this mutation. To test this hypothesis, we engrafted the SF3B1^WT^ and SF3B1^K700E^ cell lines subcutaneously into immunocompromised mice, allowed the tumors to grow, treated the mice with etoposide, olaparib, or a vehicle control and monitored tumor growth for 28 days (Fig. 4A). In keeping with the increased replication fork stalling in unperturbed SF3B1^K700E^ cells, SF3B1^K700E^ xenografts appeared to grow at a slower rate than their SF3B1^WT^ counterparts, albeit this slower growth rate did not result in significant differences in tumor volume. However, treatment with either etoposide or olaparib significantly reduced the volume of the SF3B1^K700E^-mutant tumors, but had little effect on the volume of SF3B1^WT^ tumors (Fig. 4B). In addition, in line with our previous findings, increased DNA damage (γH2AX) was observed in SF3B1^K700E^ tumors compared with SF3B1^WT^ tumors across all conditions (Supplementary Fig. S5A). These observations provide the first evidence that tumor cells harboring the SF3B1^K700E^ mutation can be selectively targeted *in vivo* using a synthetic lethality approach.

## Discussion

The spliceosome is essential to ensure the efficient and correct splicing of the majority of cellular pre-mRNAs. Consequently, most of the genes encoding components of the spliceosome are intolerant to loss-of-function mutations (29). Nevertheless, recurrent missense mutations have been identified in a number of spliceosome genes in a variety of different cancer types, suggesting a pathologic role for these mutations in tumor development (5–9). The most frequently mutated of these genes is *SF3B1*, with SF3B1^K700E^ occurring in a wide range of cancer types at varying frequencies. Despite the high frequency of *SF3B1* mutations, the functional impact of these on tumor development remains unknown. Moreover, the impact of *SF3B1* mutations on overall survival is disease dependent, for example, *SF3B1* mutations in uveal melanoma are associated with improved overall survival, while associated with poor overall survival in acute myeloid leukemia (10). However, in the context of this study, this may reflect a difference in the importance of replication stress to drive tumorigenesis or the differential dependencies on HR to maintain genome stability and cell viability following treatment between different cell types.

Whilst a number of studies have linked loss of mRNA splicing machinery components with aberrant DNA damage response (DDR) activation and/or defective DNA repair (1, 30–32), it is clear that the majority of somatically acquired mutations in spliceosome genes do not lead to a loss function. As such, it is difficult to ascertain from these studies which functions of the spliceosome complex subunits are specifically compromised by tumor-associated hypomorphic mutations. However, it has been shown that the SF3B1^K700E^ mutation alters the 3′-splice site recognition function of SF3B1, which often leads to cryptic 3′-splice site usage, resulting in loss and/or altered function of target genes and their encoded proteins. Despite this, it is still unclear as to which cellular phenotypes are specifically caused by the SF3B1^K700E^ mutation, how these might contribute to disease progression or dictate responses to therapy and which genes, whose splicing is compromised by the K700E mutation, are responsible for this phenotype. Therefore, to assess the impact of SF3B1^K700E^ mutation on the DDR, we generated an SF3B1^K700E^ cell line using CRISPR/Cas9 (16). Importantly, in keeping with the previously reported impact of SF3B1^K700E^ mutation on cryptic 3′-splice site usage, this knock-in cell line expresses a number of altered transcripts in common with those expressed in patient derived SF3B1^K700E^ tumor cells (16). More importantly, in a manner similar to the SF3B1 knockdown, cells expressing SF3B1^K700E^ display an inability to repair DSBs via an HR-dependent mechanism (33). Interestingly, unlike BRCA1 loss, the HR defect in cells with SF3B1^K700E^ mutation cannot simply be explained by an inability to promote the loading of Rad51 onto resected DSBs. However, there are a number of DNA repair genes that are misspliced and/or inefficiently exported for translation in SF3B1^K700E^ cells, including *EME1* and *XRCC3*, which both exhibit increased alternative 3′ splice site usage (Supplementary Fig. S5B and S5C). Notably, both genes have been previously shown to be important for HR downstream of Rad51 loading (34, 35) although, it remains to be determined whether the DSB repair abnormalities observed in cells expressing SF3B1^K700E^ arise directly from aberrant splicing of either of these genes.

To further complicate matters, several studies have proposed that the increased genome instability resulting from loss or mutation of components of the spliceosome, for example, SRSF2 and U2AF1 is associated with the formation of pathogenic R-loops (19, 22, 26, 30). Because the presence of R-loops is known to induce replication stress and genome instability by impeding fork progression and promoting fork collapse, it is difficult to ascertain whether the cellular phenotype caused by hypomorphic mutations in spliceosome components are a consequence of the missplicing of specific genes or just the presence of R-loops. Indeed, we also demonstrate that mutation of SF3B1 is associated with the increased presence of R-loops and that this is consistent with SF3B1^K700E^ cells displaying increased levels of spontaneously stalled forks, which can be reduced following the expression of RNaseH1. Despite this, the inability of SF3B1^K700E^ cells to protect and restart replication forks stalled with HU, or to efficiently replicate in the presence of DNA-damaging agents, known to induce replication fork reversal, cannot be adequately explained simply by an increased presence of R-loops. In line with this, the induction of RNAse-H1, which degrades R-loops, did not alter the sensitivity of SF3B1^K700E^ cells to etoposide or olaparib. In this respect, it is more plausible that these phenotypes can be explained by missplicing or reduced mRNA export of a gene(s) involved in HR. Intriguingly, unlike the other Rad51 paralogs, XRCC3 has been recently shown to be important for the restart of stalled replication forks (36). Therefore, it is conceivable that the phenotype exhibited by cells with SF3B1 dysfunction may, at least in part, be attributed to abnormal *XRCC3* gene splicing.

Interestingly, XRCC3 has also been implicated in protecting damaged replication forks from nucleolytic degradation (37), suggesting that *XRCC3* missplicing could also underlie the increased fork degradation observed in SF3B1^K700E^ cells. Although, more recently, this activity of XRCC3 has been disputed (36). Despite this, we observed that SF3B1^K700E^ cells exhibited an inability to properly recruit Rad51 to sites of stalled/damaged replication forks. However, because it is known that the HR and fork protection functions of BRCA2 are separable, our data would suggest that the fork protection defect in the SF3B1^K700E^ cells lies within the BRCA2-dependent pathway but upstream of Rad51 loading. Clearly, further investigation is needed to pinpoint the precise mechanism with which SF3B1 prevents uncontrolled nucleolytic degradation of damaged replication forks.

Importantly, irrespective of the underlying mechanism, we demonstrated that the HR and replication defects arising as a consequence of the SF3B1^K700E^ mutation can be exploited therapeutically *in vivo.* The ability of two clinically relevant antitumor therapies, etoposide and olaparib, to significantly prevent the growth of tumor xenografts harboring the SF3B1^K700E^ mutation provides compelling evidence that patients with tumors carrying this *SF3B1* mutation, or perhaps other splicing factor mutations, would be good candidates for a treatment protocol specifically tailored to target HR-deficiencies. This is particularly important for diseases such as MDS, where up to 30% of patients carry the SF3B1^K700E^ mutation. MDS generally occurs in older patients (>60 years old), whom are often not deemed fit for treatment with DNA-damaging therapies such as etoposide, which can be relatively toxic. Therefore, these patients would likely benefit from a less toxic, targeted approach, such as that offered by PARP inhibitors.

Taken together, our data demonstrate that the SF3B1^K700E^ mutation gives rise to a BRCA-like cellular phenotype and as such, indicates that the presence of this mutation and perhaps other tumor-associated spliceosome mutations could form the basis of a clinical trial to explore the efficacy of synthetically lethal inhibitors as a treatment option to specifically target these tumor cells for killing.

## Authors' Disclosures

K.I. Mills reports grants from Queen's University Belfast during the conduct of the study. D. Harkin reports grants from Medical Research Council and grants from Cancer Research UK during the conduct of the study; other support from Almac Diagnostic Services outside the submitted work. G.S. Stewart reports grants from Cancer Research UK and grants from Great Ormond Street Hospital Charity and SPARKS during the conduct of the study. K.I. Savage reports grants from Cancer Research UK and grants from Medical Research Council UK during the conduct of the study. No disclosures were reported by the other authors.

## Supplementary Material

Supplementary Data

Supplementary Table

Supplementary Table
