# Supplementary material for: Cancer-Associated SF3B1 Mutations Confer a BRCA-Like Cellular Phenotype and Synthetic Lethality to PARP Inhibitors
Source: Cancer Res. 2022 Mar 3;82(5):819–30. doi: 10.1158/0008-5472.CAN-21-1843 (PMC7612475; doi:10.1158/0008-5472.CAN-21-1843)
Supplement: Supplementary Data [file can-21-1843_supplementary_figures_suppsf.pdf]

A

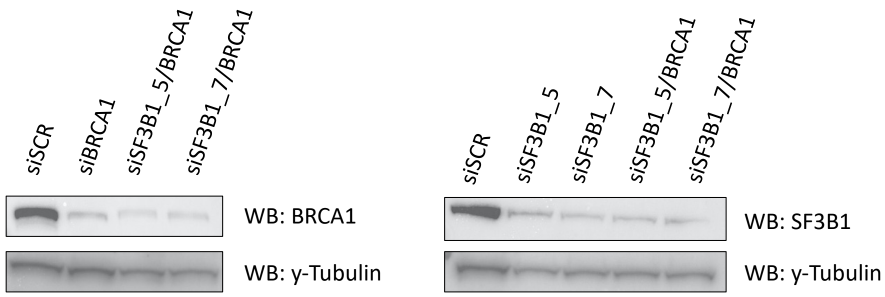

B

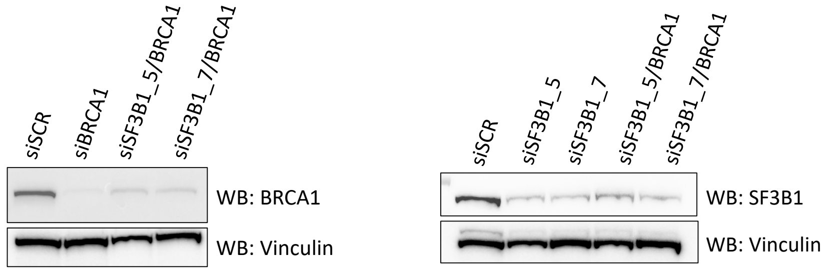

C

IPA of genes exhibiting A3SS in SF3B1^{K700E} cells

Name	p-value	Number of Molecules
RNA Post-transcriptional Modifications	2.24E^{-02} - 7.06E^{-11}	40
Protein Synthesis	1.68E^{-02} - 3.63E^{-07}	40
RNA Damage and Repair	1.42E^{-04} - 4.98E^{-07}	20
DNA Replication, Recombination and Repair	3.33E^{-02} - 1.16E^{-06}	43
Cell Cycle	3.33E^{-02} - 3.55E^{-06}	64

IPA of genes exhibiting SEs in SF3B1^{K700E} cells

Name	p-value	Number of Molecules
DNA Replication, Recombination and Repair	2.14E^{-03} - 5.31E^{-13}	121
Cell Cycle	2.22E^{-03} - 8.49E^{-13}	263
RNA Post-transcriptional Modifications	7.83E^{-04} - 2.32E^{-11}	68
Gene Expression	4.58E^{-04} - 6.35E^{-11}	248
Cell Death and Survival	2.14E^{-03} - 4.51E^{-10}	349

D

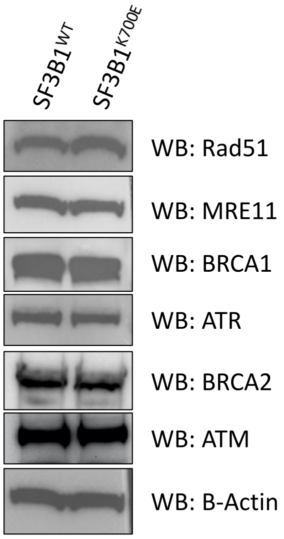

FigS1

Figure S1

A-B) Representative Western blot analysis validating the depletion of SF3B1 and BRCA1 in 293T (A) and U2OS (B) cells used to generate data presented in figure 1.

C) Ingenuity pathway analysis performed on genes with differential alternative 3' splice site usage (A3SS) (upper panel) and skipped exons (SE) (lower panel) in $SF3B1^{K700E}$ cytoplasmic fraction RNA-seq data compared to their $SF3B1^{WT}$ counterparts. Top 5 deregulated pathways are highlighted, along with number of molecules within each pathway deregulated in $SF3B1^{K700E}$ cells and associated enrichment p-values.

D) Representative Western blot analysis of indicated HR proteins in $SF3B1^{WT}$ and $SF3B1^{K700}$E cells.

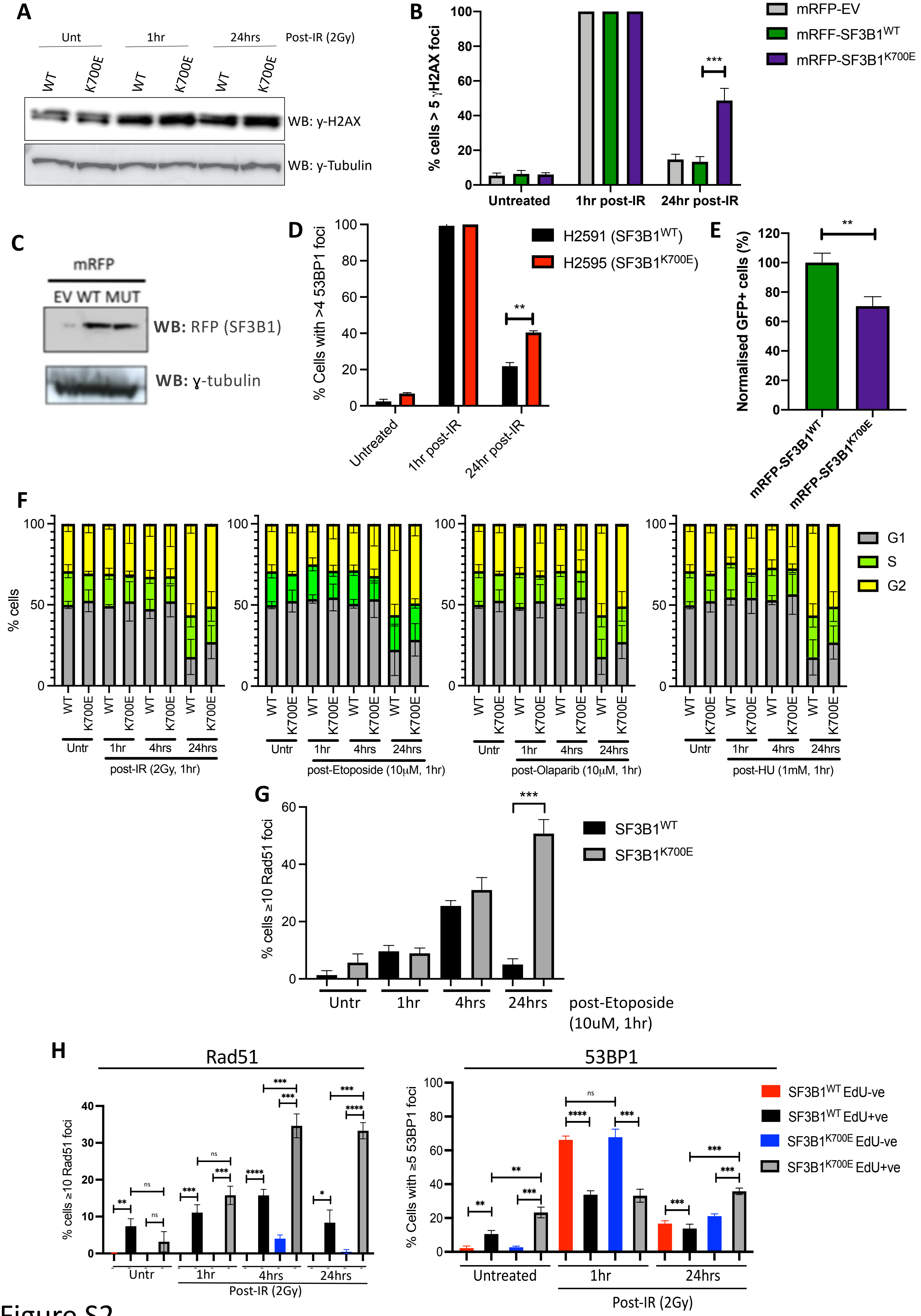
A
Unt
1hr
24hrs
Post-IR (2Gy)
WT
K700E
WB: γ-H2AX
WB: γ-Tubulin
B
% cells > 5 γH2AX foci
Untreated
1hr post-IR
24hr post-IR
mRFP-EV
mRFF-SF3B1WT
mRFP-SF3B1K700E
C
mRFP
EV WT MUT
WB: RFP (SF3B1)
WB: γ-tubulin
D
% Cells with >4 53BP1 foci
H2591 (SF3B1WT)
H2595 (SF3B1K700E)
Untreated
1hr post-IR
24hr post-IR
E
Normalised GFP+ cells (%)
mRFP-SF3B1WT
mRFP-SF3B1K700E
F
% cells
G1
S
G2
Untr
1hr
4hrs
24hrs
post-IR (2Gy, 1hr)
post-Etoposide (10μM, 1hr)
post-Olaparib (10μM, 1hr)
post-HU (1mM, 1hr)
G
% cells ≥10 Rad51 foci
SF3B1WT
SF3B1K700E
Untr
1hr
4hrs
24hrs
post-Etoposide (10uM, 1hr)
H
Rad51
53BP1
% cells ≥10 Rad51 foci
% Cells with ≥5 53BP1 foci
Untr
Untreated
1hr
4hrs
24hrs
Post-IR (2Gy)
SF3B1WT EdU-ve
SF3B1WT EdU+ve
SF3B1K700E EdU-ve
SF3B1K700E EdU+ve

Figure S2

Figure S2

A) Representative Western blot showing γ-H2AX levels in untreated SF3B1^{WT} (WT) or SF3B1^{K700E} (K700E) cells and 1 and 24hrs following IR (2Gy).

B) Quantification of γ-H2AX foci in U2OS cells transfected with mRFP empty vector (mRFP-EV) or mRFP-SF3B1^{WT} or mRFP-SF3B1^{K700E} 1 and 24-hours following IR (2Gy). Quantification was carried out from three independent experiments (≥100 cells were scored/experiment). Mean percent cells containing ≥5 γ-H2AX foci is plotted ± SEM. Significant differences in the fraction of cells containing ≥5 γ-H2AX foci were assessed using two-tailed t-test and are indicated by ∗∗∗p < 0.001.

C) Representative Western blot showing ectopic expression of WT and K700E mutant mRFP-SF3B1 in U2OS cells used for DNA repair assays shown in B.

D) Quantification of 53BP1 foci in untreated H2591 (SF3B1^{WT}) and H2595 (SF3B1^{K700E}) mesothelioma cells and 1 and 24-hours following IR (2Gy). Quantification was carried out from three independent experiments (≥100 cells were scored/experiment). Mean percent cells containing ≥4 53BP1 foci is plotted ± SEM. Significant differences in the fraction of cells containing ≥4 53BP1 foci were assessed using two-tailed t-test and are indicated by ∗∗∗p < 0.001.

E) Quantification of GFP positive cells by flow cytometry from a DR-GFP reporter cassette stably integrated in U2OS cells transfected with mRFP-SF3B1^{WT} or mRFP-SF3B1^{K700E} to assess HR. Mean % GFP positive cells normalised to mRFP-SF3B1^{WT} transfected cells is plotted ± SEM. Significant differences in Mean % GFP positive cells was assessed by two-tailed t-test. **p<0.01.

F) Propidium Iodide flow cytometric evaluation of cell cycle stage, in untreated SF3B1^{WT} and SF3B1^{K700E} cells or following treatment with IR (2Gy) or 1 hour Etoposide (10 µM), Olaparib (10 µM), or HU (1 mM) after which cells were allowed to recover for the indicated time points. Mean cell cycle fraction from three independent experiments (10,000 cells/experiment) is plotted ± SEM. Significance between cell cycle stage in SF3B1^{WT} and SF3B1^{K700E} cells was assessed using two-way ANOVA, with Tukey's multiple comparisons test but no significant differences were observed.

G) Quantification of Rad51 foci in untreated SF3B1^{K700E} and SF3B1^{WT} cells and 1, 4 and 24 hr following 1hr of Etoposide (10 µM) treatment, from three independent experiments (≥100 cells were scored/experiment). Mean percentage of cells containing ≥10 Rad51 foci is plotted ± SEM. Significant differences in the fraction of cells containing ≥10 Rad51 foci were assessed using two tailed t-tests. ****p< 0.0001.

H) Quantification of Rad51 foci (left) and 53BP1 foci (right) in untreated SF3B1^{K700E} and SF3B1^{WT} cells and 1, 4 or 24 hr following IR (2Gy) treatment, from three independent experiments (≥100 cells were scored/experiment). Prior to treatment, cells were pulse labelled

with 10 μM EdU for 30 mins and EdU stained using Click-It chemistry, allowing quantification of Rad51/53BP1 foci in EdU negative and positive cells. Mean percentage of cells containing ≥10 Rad51 or ≥5 53BP1 foci is plotted ± SEM. Significant differences in the fraction of cells containing ≥10 Rad51 foci were assessed using multiple two-tailed t-tests, with Holm-Šidák multiple comparisons test and is indicated by *$p<0.05$, **$p<0.01$, ***$p<0.001$, ****$p<0.0001$.

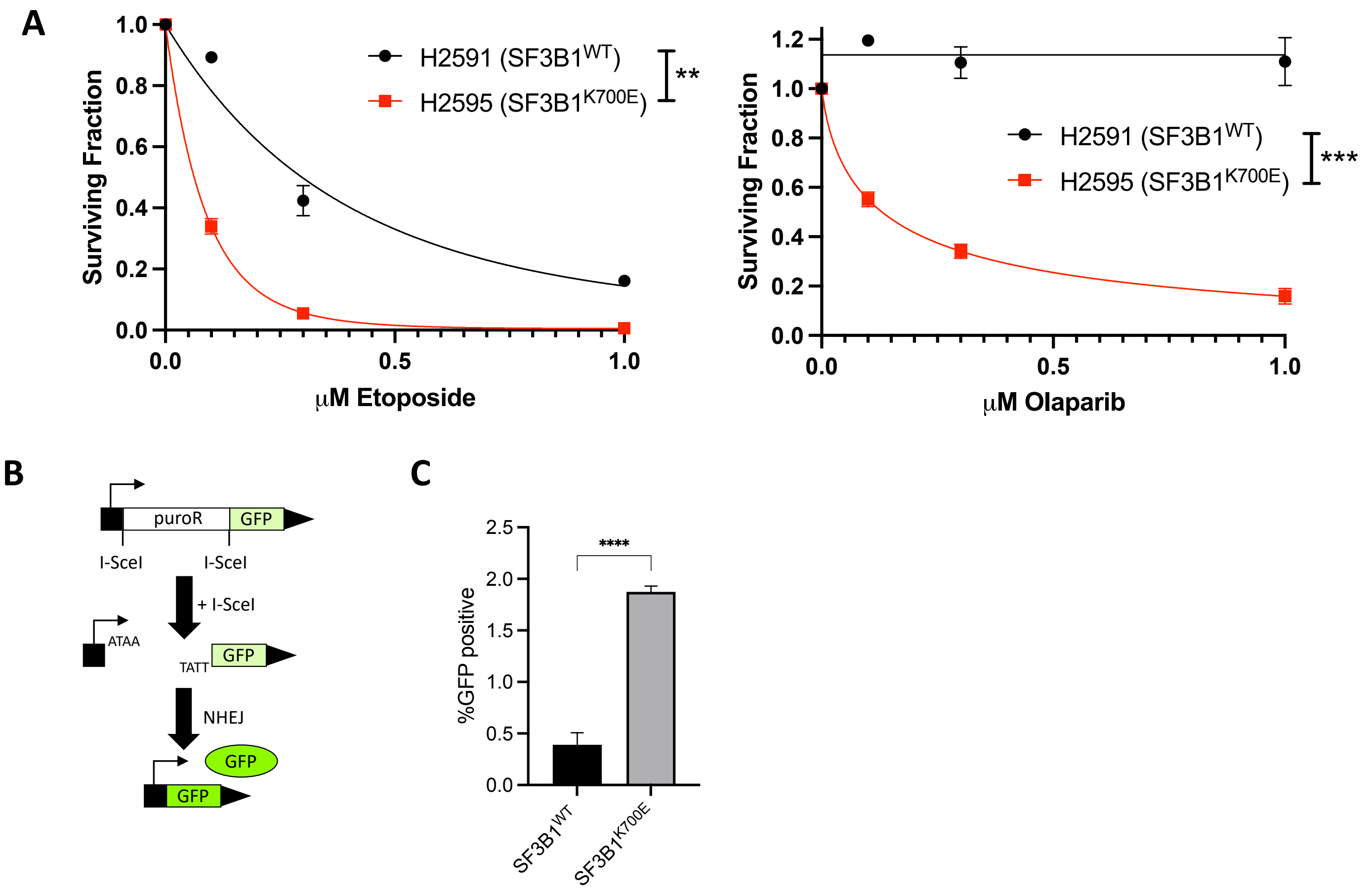
A
Surviving Fraction
µM Etoposide
H2591 (SF3B1WT)
H2595 (SF3B1K700E)
**
Surviving Fraction
µM Olaparib
H2591 (SF3B1WT)
H2595 (SF3B1K700E)
***
B
puroR
GFP
I-SceI
I-SceI
+ I-SceI
ATAA
TATT
GFP
NHEJ
GFP
GFP
C
%GFP positive
****
SF3B1WT
SF3B1K700E

Figure S3

Figure S3

A). Clonogenic survival assays in H2591 ($SF3B1^{WT}$) and H2595 ($SF3B1^{K700E}$) mesothelioma cells with increasing doses of Etoposide (left panel) or Olaparib (right panel) demonstrating increased sensitivity to etoposide and olaparib in H2595 ($SF3B1^{K700E}$) compared to H2591 ($SF3B1^{WT}$) cells. Mean surviving fraction of three independent experiments is plotted ± SEM. Significant differences in survival were assessed using two-tailed t-test and are indicated by ∗∗p<0.01, ***p<0.001.

B) Schematic representation of the EJ-5 NHEJ reporter system used in (H). Briefly, the GFP gene is separated from the CMV promoter by a puromycin resistance gene (puroR) flanked by two I-Sce I restriction sites. Ectopic expression of I-Sce I results in removal of the puromycin cassette and in a fraction of cells, NHEJ results in the ligation of the GFP gene adjacent to the CMV promoter, resulting in GFP expression.

C) Quantification of GFP positive cells by flow cytometry from a EJ-5 reporter cassette (G) stably integrated in $SF3B1^{WT}$ or $SF3B1^{K700E}$ K562 cells, to assess NHEJ. Mean % GFP positive cells from three independent experiments (10,000 cells/experiment) is plotted ± SEM. Significant differences in % GFP positive cells was assessed using two-tailed t-tests and is indicated by ****p<0.0001.

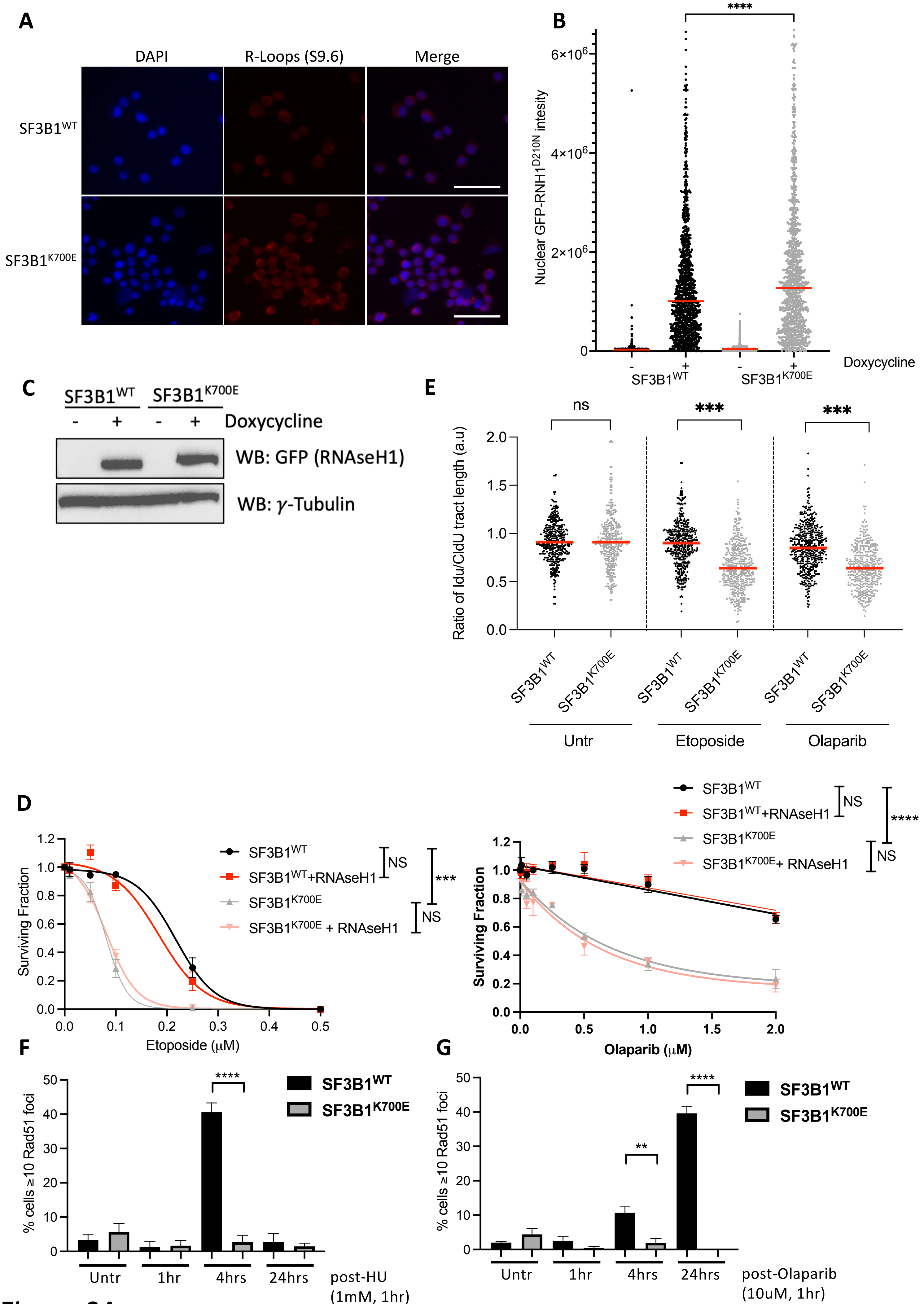
A
DAPI
R-Loops (S9.6)
Merge
SF3B1WT
SF3B1K700E
B
****
Nuclear GFP-RNH1D210N intesity
6×10^6
4×10^6
2×10^6
0
-
+
SF3B1WT
SF3B1K700E
Doxycycline
C
SF3B1WT
SF3B1K700E
- + - +
Doxycycline
WB: GFP (RNAseH1)
WB: γ-Tubulin
E
ns
***
***
Ratio of Idu/CldU tract length (a.u)
2.0
1.5
1.0
0.5
0.0
SF3B1WT
SF3B1K700E
Untr
Etoposide
Olaparib
D
1.2
1.0
0.8
0.6
0.4
0.2
0.0
Surviving Fraction
SF3B1WT
SF3B1WT+RNAseH1
SF3B1K700E
SF3B1K700E + RNAseH1
NS
***
NS
0.0
0.1
0.2
0.3
0.4
0.5
Etoposide (μM)
SF3B1WT
SF3B1WT+RNAseH1
SF3B1K700E
SF3B1K700E+ RNAseH1
NS
****
NS
Surviving Fraction
0.0
0.5
1.0
1.5
2.0
Olaparib (μM)
F
50
40
30
20
10
0
% cells ≥10 Rad51 foci
****
SF3B1WT
SF3B1K700E
Untr
1hr
4hrs
24hrs
post-HU
(1mM, 1hr)
G
% cells ≥10 Rad51 foci
**
****
SF3B1WT
SF3B1K700E
Untr
1hr
4hrs
24hrs
post-Olaparib
(10uM, 1hr)

Figure S4

Figure S4

A) Representative immunofluorescent staining of R-loops (S9.6) in untreated SF3B1^{WT} and SF3B1^{K700E} K562 cells. White bar indicates 100 µm.

B) Quantification of nuclear fluorescence intensity from SF3B1^{WT} and SF3B1^{K700E} cells with and without induction of D210N GFP-RNAseH1 (1 µg/mL Doxycycline, 24 hrs). D210N GFP-RNAseH1 binds R-loops but cannot resolve them. Cells were detergent extracted prior to fixation to remove non-bound D210N GFP-RNAseH1, prior to nuclear fluorescence quantification via high content imaging.

C) Representative Western blot showing induction of GFP-RNAseH1 in SF3B1^{WT} and SF3B1^{K700E} cells following 24hrs induction with 1µg/mL Doxycycline. Cells where stably infected with inducible GFP-RNAseH1.

D) Clonogenic survival assays in SF3B1^{K700E} and SF3B1^{WT} cells, treated with increasing doses of etoposide (left panel) or olaparib (right panel) with, or without, induction of RNaseH1 (1 µg/mL Doxycycline). Mean surviving fraction +/- SEM from three independent experiments plotted. Significant differences (two-way ANOVA, with Tukey's multiple comparisons test) between conditions is indicated, ***p< 0.001, ****p<0.0001.

E) Replication fork speed in untreated or Etoposide (50 nM, 2hrs) or Olaparib (10 µM, 2hrs) treated SF3B1^{WT} and SF3B1^{K700E} cells. Data points represent fork speed from three independent experiments with median fork speed for each condition indicated (red line). Significant differences in fork speed were assessed using unpaired Mann-Whitney U test. ***p< 0.001.

F-G) Quantification of Rad51 foci in untreated SF3B1^{K700E} and SF3B1^{WT} cells and 1, 4 and 24 hr following 1hr of D) Hydroxyurea (1mM), or E) Olaparib (10µM) treatment, from three independent experiments (≥100 cells were scored/experiment). Mean percentage of cells containing ≥10 Rad51 foci is plotted ± SEM. Significant differences in the fraction of cells containing ≥10 Rad51 foci were assessed using two tailed t-tests. ****p< 0.0001.

A

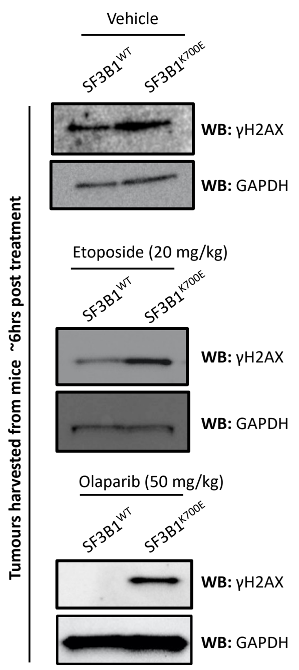

B

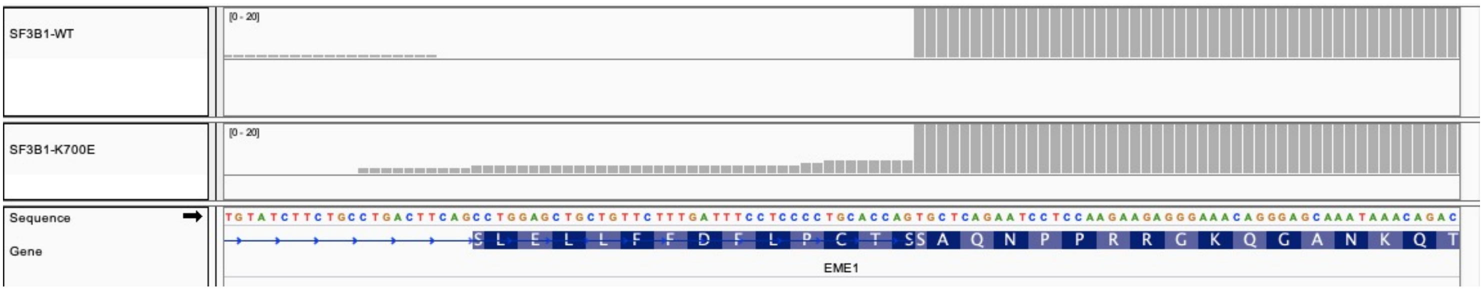

C

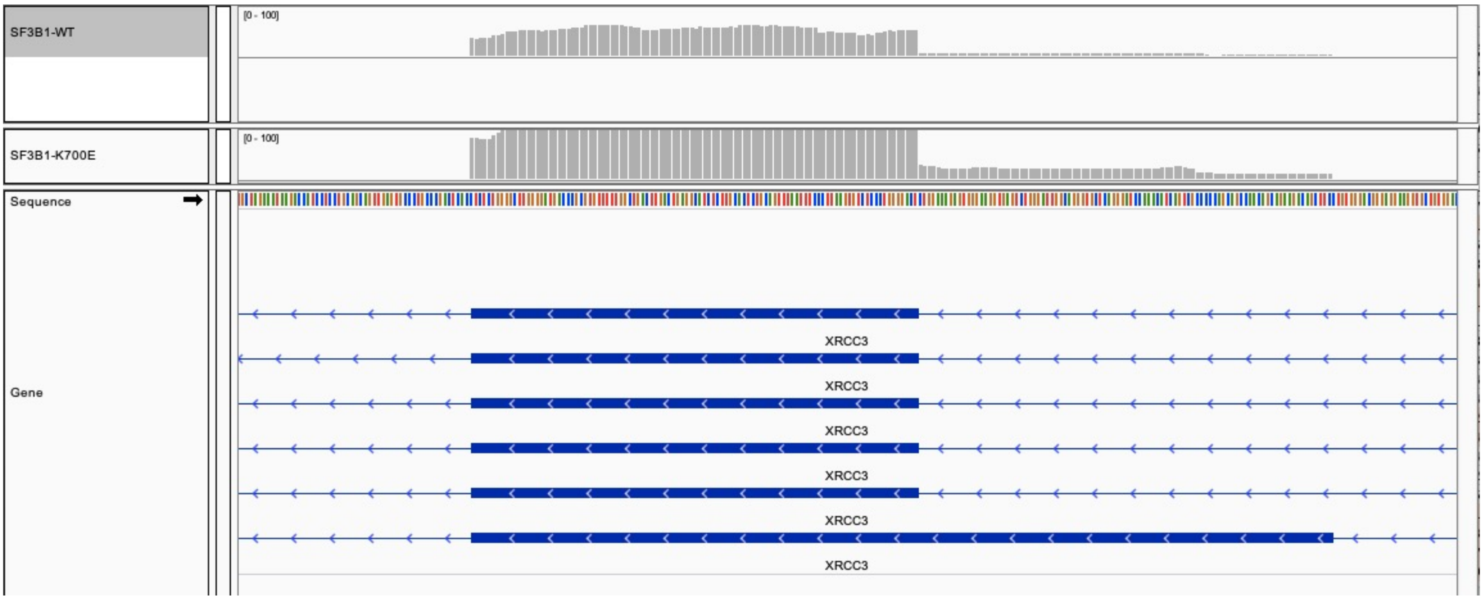

Figure S5

Figure S5

A) Western blot of γ-H2AX in SF3B1^{WT} or SF3B1^{K700E} xenograft tumours harvested from mice 6hours after treatment as indicated.

B-C) Total normalized RNA-seq read counts generated from cytoplasmic RNA fractions harvested from SF3B1^{WT} and SF3B1^{K700E} cells (16). Coverage plots at EME1 exon 6 and XRCC3 exon 3 showing alternative 3′ splice site usage at these exons in SF3B1^{K700E} cells compared to SF3B1^{WT}.
